# Targeting type I interferons in systemic lupus erythematous

**DOI:** 10.3389/fphar.2022.1046687

**Published:** 2023-01-16

**Authors:** Sebastian Bruera, Thandiwe Chavula, Riya Madan, Sandeep K. Agarwal

**Affiliations:** ^1^ Section of Immunology, Allergy and Rheumatology, Department of Medicine, Baylor College of Medicine, Houston, TX, United States; ^2^ Section of General Internal Medicine, Department of Medicine, Baylor College of Medicine, Houston, TX, United States

**Keywords:** lupus (SLE), interferons, rheumatology, dendritic cell, treatment

## Abstract

Systemic lupus erythematosus (SLE) is a complex autoimmune disease with systemic clinical manifestations including, but not limited to, rash, inflammatory arthritis, serositis, glomerulonephritis, and cerebritis. Treatment options for SLE are expanding and the increase in our understanding of the immune pathogenesis is leading to the development of new therapeutics. Autoantibody formation and immune complex formation are important mediators in lupus pathogenesis, but an important role of the type I interferon (IFN) pathway has been identified in SLE patients and mouse models of lupus. These studies have led to the development of therapeutics targeting type I IFN and related pathways for the treatment of certain manifestations of SLE. In the current narrative review, we will discuss the role of type I IFN in SLE pathogenesis and the potential translation of these data into strategies using type I IFN as a biomarker and therapeutic target for patients with SLE.

## Introduction

Systemic Lupus Erythematosus (SLE) is a complex autoimmune disease clinically characterized by inflammation of tissues clinically manifesting as rashes, inflammatory arthritis, serositis, glomerulonephritis, central nervous system involvement and hematological abnormalities. SLE predominates in women of childbearing age with a 9:1 female to male prevalence and higher incidence in black and Hispanic women (McCarty et al., 1995; Izmirly et al., 2017; Izmirly et al., 2021). Genome Wide Association Studies (GWAS) have been key in identifying SLE susceptibility genes in different ancestral populations (Hom et al., 2008; International Consortium for Systemic Lupus Erythematosus et al., 2008; Sánchez et al., 2011; Sanchez et al., 2011; Bentham et al., 2015; Wang et al., 2021). Other factors including environmental, hormonal, and infections also promote risk of disease development (James et al., 2001; Costenbader et al., 2007; Cooper et al., 2010).

Therapeutic approaches to SLE patients depend on the disease manifestations and activity, but ultimately seek to achieve remission or low disease activity using a variety of immunomodulatory and immunosuppressive therapies. Commonly used therapeutics include hydroxychloroquine, methotrexate, azathioprine, mycophenolate mofetil, and cyclophosphamide. Treatment paradigms in SLE is now entering the age of biologics targeting specific cellular targets and cytokines. Belimumab, a monoclonal antibody that targets B Lymphocyte stimulator (BLys) and inhibits the survival of autoreactive B cells, is approved for use in a variety of manifestations of SLE including lupus nephritis (Baker et al., 2003; Furie et al., 2011; Navarra et al., 2011). Based on extensive preclinical and clinical studies further detailed in this review, the type I interferon (IFN) pathway has now become a new therapeutic target (Fure et al., 2019; Morand et al., 2020).

Immunologically, SLE is characterized by activation of aberrant autoreactive T and B cell activation, autoantibody production against nuclear antigens and immune complex formation. In addition, innate immunity is central to the immunopathogenesis of SLE. Much attention has focused on type I IFN production by plasmacytoid dendritic cells (pDCs) which activates multiple immune cells and is central in B cell activation and autoantibody production (Choi et al., 2012; Pan et al., 2020). There is growing interest in the therapeutic targeting of type I IFNs in SLE. In this review, we will discuss the role of type I IFNs in SLE immunopathogenesis starting with a basic overview of type I interferons and murine models of lupus. We will also discuss translational studies in SLE patients that demonstrate the importance of type I interferons in SLE and finally discuss the more recent clinical trials that led to the use of therapeutics targeting type I interferons being used in the clinics for the management of SLE.

## Biology and function of type I interferons

Type I IFNs are a family of cytokines that mediate responses to antiviral infection. Discovered in 1957 as the initial defense to viral infections (Isaacs and Lindenmann, 1957), our understanding has expanded with numerous studies implicating their involvement in cell growth regulation, immune cell activation, bacterial responses, and autoimmunity. Type I IFNs also possess anti-tumor properties including immune-editing, inhibition of cell growth, and angiogenesis (Yano et al., 1999; Dunn et al., 2005; Swann et al., 2007). The IFN-I locus in humans codes for IFN-α (13 subtypes), IFN-β, IFN-ε, IFN-ω, and IFN-κ. This review focuses on IFN-α and IFN-β. The production of IFN-α is predominant in dendritic cells (DCs), with pDCs being the most potent producers (Cella et al., 1999; Siegal et al., 1999). Myeloid DCs, monocytes and macrophages also produce IFN-α albeit at lower levels (Izaguirre et al., 2003). IFN-β is encoded by a single *IFNβ* gene and is mostly produced by fibroblasts and epithelial cells (Reis et al., 1989; Erlandsson et al., 1998).

### Type I IFN signaling

**FIGURE 1:**
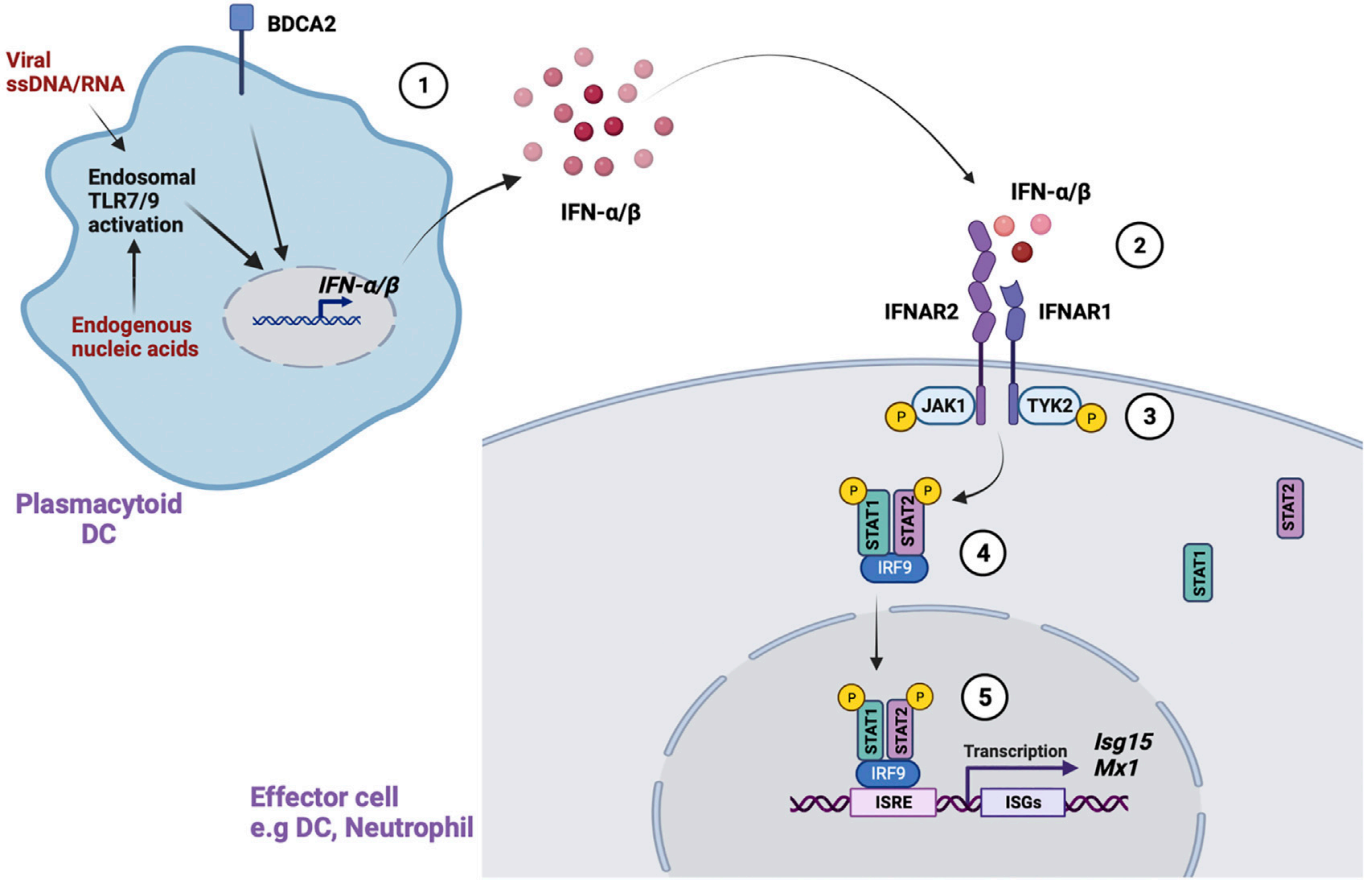
Type 1 IFN signaling. Type 1 IFN signaling is mediated through several steps. (McCarty et al., 1995). Activation of endosomal TLR7/9 by viral ssDNA/RNA or endogenous nucleic acids in pDCs leads to IFN-α/β production. Activation of an important receptor, BDCA2, also promotes IFN-α/β production (Izmirly et al., 2017). In the canonical pathway, IFN-α/β binds to the IFN-α receptor (IFNAR) in IFN responsive cells (Izmirly et al., 2021). This leads to phosphorylation and activation of IFNAR associated JAKs (JAK1, Tyk2) (International Consortium for Systemic Lupus Erythematosus et al., 2008). The activated JAKs phosphorylate STAT1/STAT2, which form a heterodimer that further complexes with IRF9 in the cytoplasm (Hom et al., 2008). Finally, the STAT1-STAT2-IRF9 complex translocates to the nucleus, binds to conserved IFN-stimulated response elements (ISREs), and leads to transcription of IFN stimulated genes (ISGs).

All type I IFNs bind a common receptor called IFN-α receptor (IFNAR) (Figure 1). IFNAR is a transmembrane receptor consisting of two subunits, IFNAR1 (Uzé et al., 1990) and IFNAR2 (Novick et al., 1994) that interact with a group of kinases called Janus activated kinases (JAKs) in the cytoplasm. IFNAR1 constitutively associates with tyrosine kinase 2 (TYK2) whereas IFNAR2 associates with JAK1 (Velazquez et al., 1992; Domanski et al., 1995). These JAKs activate a group of transcription factors called signal transducer and activator of transcription (STAT). Extensive complementary work by James Darnell, Ian Kerr and George Stark established the JAK-STAT signaling as the major pathway through which type 1 IFNs mediate their effects (Darnell et al., 1994). Binding of IFN-α/β to the IFNAR results in auto-phosphorylation and activation of the IFNAR-associated JAKs, which in turn phosphorylate and activate STATs. The phosphorylated STATs form heterodimers or homodimers that translocate to the nucleus to induce transcription of IFN stimulated genes (ISGs).

In the canonical pathway, the phosphorylated STAT1-STAT2 heterodimer forms an important complex with IFN-regulatory factor 9 (IRF9) called IFN-stimulated gene factor 3 (ISGF3) in the cytosol (Fu et al., 1992). The ISGF3 complex translocates to the nucleus and binds conserved elements called IFN-stimulated response elements (ISREs) in ISGs to initiate transcription of antiviral and antibacterial genes. IFN-α/β can also signal through non-canonical pathways involving STAT1 homo dimers that directly translocate to the nucleus and bind to γ-activated sequences (GAS) in gene promoters. GAS sequences are commonly used by IFN-γ signaling. IFN-α/β can also signal through other STATs, including STAT3, STAT4 and STAT5, which associate with cytokine signaling pathways. The non-canonical IFN signaling pathways and their effects have been extensively detailed in other reviews (Saleiro and Platanias, 2019; Mazewski et al., 2020).

The effects of type I IFN signaling are broad. At the transcription level, hundreds of ISGs induced by IFN-α/β have been identified through microarrays and more recently, RNA sequencing assays. Early work by Der et al identified 122 ISGs using oligonucleotide microarrays. The human fibrosarcoma cell line HT1080 was induced by IFN-α, IFN-β and IFN-γ. Of the 122 ISGs, 40 were known and 82 were novel genes that regulated apoptosis, angiogenesis, protein kinase receptors, and synthetases, among others (Der et al., 1998). De Veer et al extended these findings in a subsequent review of different microarray analysis data. Their analysis detailed over 300 genes that regulate cellular functions including adhesion, apoptosis, immune modulation, and cell growth (de Veer et al., 2001).

The number of ISGs discovered has grown exponentially over time with studies expanding to different primary cells and cell lines. These include PBMCs, human and murine epithelial cells, fibroblasts, T cells, and DCs (Hilkens et al., 19502003) (Indraccolo et al., 2007) (Rani et al., 2007). Recently, 617 ISGS with 137 novel genes were identified in three human immune cell lines, CD4^+^ T cell-derived CEM, monocyte-derived U937 and B cell-derived Daudi cells after stimulation with IFN-α (Zhang et al., 2018).

At cellular level, IFN-α/β signaling affects both innate and adaptive immune cells, with cell specific functions. DCs are a key source and effector of type I IFNs. IFN-α has autocrine effects on pDCs, with increased IFN-α secretion in response to type I IFN signaling (Montoya et al., 2002). Further, IFN-α promotes DC maturation, expression of costimulatory MHCII molecules and antigen presentation (Simmons et al., 2012). In macrophages, IFN-α enhances LPS-induced activation, cell survival, and Mtb-induced cell death in murine bone marrow derived macrophages *in vitro* (Vadiveloo et al., 2000; Zhang et al., 2020). In Natural Killer (NK) cells, IFN-α/β promotes NK cell cytotoxicity (Orange and Biron, 1996; Nguyen et al., 2002). In T-cells, IFN-α/β promotes survival of activated CD4^+^ and CD8^+^ T cells *in vitro* (Marrack et al., 1999), CD8^+^ T cells differentiation and expansion (Curtsinger et al., 2005), and clonal expansion of CD4^+^ and CD8^+^ T cells in response to viral infection *in vivo* (Kolumam et al., 2005). Finally, B cells have enhanced activation, antibody production, and class switching *in vivo* in response to IFN-α (Coro et al., 19502006).

### Type I IFNs in SLE pathogenesis

Although type I IFNs have a central role in anti-viral responses, their effects in regulating the immune system are crucial in autoimmune disease. A pivotal study by Steinberg et al was the first to establish a link between type I IFNs and SLE in 1969. NZB/W F1 mice, which spontaneously develop lupus, were treated with polyinosinic: polycytidylic acid (Poly I: C), a synthetic double stranded RNA with antiviral properties, to ameliorate disease. However, the mice produced significant amounts of IFN which contributed to worsening disease (Steinberg et al., 1969). Further work in different genetic models has extended these observations. In MRL/lpr mice, a different spontaneous lupus model, injection of Poly I:C led to accelerated glomerulonephritis and autoantibody production (Braun et al., 2003). Conversely, type I IFN receptor deficiency attenuated disease in both spontaneous (Braun et al., 2003; Santiago-Raber et al., 2003; Keller et al., 2021) and induced models of lupus (Nacionales et al., 2007). Thus, a pathogenic role of type I IFNs in murine lupus *in vivo* was established.

Alterations in DCs have been linked to IFN-α production in pediatric SLE patients (Blanco et al., 2001). IFN-α mediated differentiation of monocytes to DCs and the induction of DCs correlated with increased serum IFN-α production in SLE (Blanco et al., 2001). Further evidence demonstrated that type 1 IFN production in SLE is mainly driven by pDCs. In support of this, depletion, or genetic impairment of pDCs *in vivo* has been shown to ameliorate disease and IFN-α/β driven inflammation in murine models (Rowland et al., 2014; Sisirak et al., 2014). In humans, targeting of pDCs with a monoclonal antibody to blood dendritic cell antigen 2 (BDCA2) receptor reported decreased expression of IFN-regulated genes and reduced immune infiltrates in skin lesions of SLE patients with cutaneous disease (Furie et al., 2019).

Several factors can activate pDCs in SLE. Endogenous nucleic acids released from apoptotic or necrotic cells can form immune complexes (ICs) with IgG autoantibodies (Båve et al., 2000; Lövgren et al., 2004). Uptake of these ICs by pDCs leads to activation of endosomal toll-like receptors TLR7 and TLR9, which triggers IFN-α production. Another important trigger is Neutrophil Extracellular Traps (NETs) where neutrophils extrude their nucleic material in an extracellular web-like structure. SLE patients have increased formation of NETs coupled with dysfunctional clearance of these NETs, resulting in their exposure to autoreactive B cells and autoantibodies (Lande et al., 2011). Indeed, studies have shown that NETs are important in driving pDC activation and IFN-α production *in vitro* (Garcia-Romo et al., 2011). Finally, pDCs can also be activated by viral or bacterial infections in a TLR7-MYD88 dependent pathway (Abbas et al., 2020). In addition to pDCs, other cells including monocytes and neutrophils have been shown to produce IFN-α in SLE (Stone et al., 2012; Palanichamy et al., 2014). However, the extent of their contribution in the clinical setting has not been fully established and more work still needs to be done.

SLE patients have high levels of IFN-α (Niewold et al., 2007). However the large number of type I IFN, the complexity of IFN signaling and the myriad of upregulated ISGs in SLE presents a unique challenge in pinpointing suitable targets. Discovery of the “IFN signature”, a cluster of upregulated ISGs, has overcome this challenge in part and enabled better assessment of IFN expression in the clinical setting (Baechler et al., 2003). It is now established that 50–70% of adult and pediatric SLE patients have an IFN signature that correlates with disease activity and severity (Wahadat et al., 2018) as will be further discussed in later sections.

### Type I IFN in murine models of lupus

The bulk of our knowledge on type I IFN dysregulation in SLE is driven by *in vivo* work. In NZB/W F1 mice susceptible to lupus, continuous *in vivo* administration of adenovector-derived murine IFN-α resulted in enhanced clinical disease and IFN signaling (Mathian et al., 2005; Liu et al., 2011). In comparison, other spontaneous lupus models including MRL/lpr, MRL^+/+^, and B6/lpr have a weak or absent IFN signature (Zhuang et al., 2015). A possible explanation for these differences is the different genetic background of these models and the spontaneous *lpr* mutation they carry which accelerates lymphoproliferation but could also influence type 1 IFN signaling. More work still needs to be done to parse this out.

The pristane induced lupus model is an established model with strong type 1 IFN signaling and clinical features that mimic human disease in non-autoimmune-prone mice (Reeves et al., 2009). Interestingly, IFN production appears to be largely driven by Ly6C + inflammatory monocytes in a TLR7-dependent manner, and less so by pDCs (Lee et al., 2008). Utility of pristane induction has also allowed for the identification of individual factors that regulate IFN signaling in SLE pathogenesis. In a novel humanized lupus mouse model, injection of pristane into mice reconstituted with human immune system mimics human disease with autoantibody production, inflammatory cytokines and enhanced ISGs in hepatocytes (Gunawan et al., 2017). Similar to the pristine induced lupus model, cutaneous administration of the TLR7 agonist Imiquimod in different strains of wild-type mice induced pDC-driven lupus-like disease with glomerulonephritis, autoantibodies, and increased expression of the IFN regulated genes *Mx1* and *IFna* (Yokogawa et al., 2014). Expanding on this, Liu et al demonstrated enhanced clinical disease that was TLR9 mediated (Liu et al., 2018).

### Genetic association between type1 IFN pathway and SLE susceptibility

The development of SLE is strongly influenced by genetic factors. The human leukocyte antigen region (HLA) is the strongest predictor, but the genetic component of disease susceptibility in autoimmune disease, in particular SLE is complex involving large number of variants and loci across the genome (Ortíz-Fernández et al., 2022). While monogenic causes of SLE (e.g., complement deficiencies) exist, the genetic architecture of SLE is far more complex but have provided insight into the immunopathogensis of SLE, especially with regards to type I IFN. Genome wide association studies in SLE patients has demonstrated single nucleotide polymorphisms (SNPs) in loci near IFN related genes, in particular interferon regulatory factor 5 (IRF5), IRF7, IRF8, STAT4 and Tyk2, that are associated with the risk of developing SLE in adults (Sigurdsson et al., 2005; Graham et al., 2007). Consistent with these observations, using a candidate gene approach of type I IFN related genes, the T allele of rs3747517 in the IFIH1 gene was associated with a reduced risk of SLE while the T allele of rs7574865 in STAT4 was associated with an increased risk of developing SLE in children and adolescents (Zedan et al., 2021). In conjunction with our understanding of type I IFN in mouse models with SLE and translational studies demonstrating increased expression of type I IFN and related pathways in SLE, these genetic studies strongly support an important role for type I IFN related pathways in SLE pathogenesis and the potential for translation of these findings to the care of patients with SLE.

In summary, extensive research has established the role of type1 IFNs in SLE at genetic, molecular, and cellular level. Type I interferons in pDCs are an important driver of disease. Given the complexity of type 1 IFN signaling and the challenges with elucidating mechanisms in human SLE patients, it not surprising that a significant amount of the supportive evidence is derived from both spontaneous and induced murine models of SLE. Later in this review, we will discuss the evidence for type I interferon activation in SLE patients and review the clinical trails that have led to type I interferon directed therapies in SLE.

## Clinical correlations between type I IFN and SLE

### IFN therapy and lupus-like illnesses

The use of IFN therapy for cancer and viral infections, such as hepatitis B and C, contributed to the earliest clinical evidence that IFN may play a role in the development of SLE. In the 1990s and early 2000s, multiple cases of autoimmune diseases arising shortly after administration of IFN therapy were reported (the most common of which was thyroid disease). Multiple cases of new diagnoses of SLE after IFN therapy have been reported (García-Porrúa et al., 1998; Wilson et al., 2002; Khalil and Khokhar, 2016). It is unclear whether these patients may have had “pre-clinical” SLE that was exacerbated by IFN therapy *versus* new formulation of antibodies. One study showed that 61% of patients that were initially antinuclear antibody (ANA) negative developed positive ANA tests after IFN therapy for hepatitis C (Noda et al., 1996). Although this study did not show causality, it highlighted a potential association between IFN therapy and development of autoimmunity.

### Association of baseline type I IFN activity with disease activity and clinical manifestations

After the discovery that dysregulation of the type I IFN pathways may contribute to the pathogenesis of SLE, there have been multiple cross-sectional and prospective studies seeking to determine if increased type I IFN levels and/or the type I IFN signature correlates with disease activity. There is a strong correlation between baseline increased IFN levels and SLE disease activity. Table 1 summaries a large number of published studies demonstrating an association of increased baseline type I IFNs or type I IFN signature levels with increased SLE disease activity in patients with new or established SLE (Al-Mutairi et al., 2007; Fu et al., 2008; Landolt-Marticorena et al., 2009; Willis et al., 2012; Rose et al., 2013; Schneider et al., 2015; Munroe et al., 2017; Liu et al., 2018; Petri et al., 2019; Adel and Sadeq, 2020; Guthridge et al., 2020; Mai et al., 2021). One notable study followed 137 SLE patients for 5 years after initial IFN signatures were obtained (Mai et al., 2021). The type I IFN signature was determined based on the expression of five IFN related genes, *EPSTI1, IFI44L, LY6E, OAS3,* and *RSAD2*. Patients with high type I IFN signatures were more likely to have more severe disease activity as defined by the SLE disease activity-2000 index (SLEDAI-2K) at study entry and over time, including an increase in disease flares and a need for treatment with additional immunosuppressive agents and corticosteroids. In contrast, patients with lower type I IFN signatures were more likely to achieve low lupus disease activity. Northcott et al analyzed 729 serum samples from 205 SLE patients, including 142 patients with multiple samples (Northcott et al., 2022). High baseline type I IFN signatures were observed in 63% of patients and predicted future high disease activity in multiple organ domains but the type I IFN signature itself was stable and did not change with disease activity (Northcott et al., 2022). Finally, prospective studies have also shown that an increased baseline IFN is associated with an increased risk of disease flare in the future (Merrill et al., 2017). These studies show that baseline increases in type I IFN signatures may have potential prognostic value and predict severe disease activity.

**TABLE 1:** Selected studies with clinical correlations of interferon signature and SLE.

Author	Country	Type of Study, N	IFN measured	Clinical outcomes	Summary of findings
Adel 2020 (Adel and Sadeq, 2020)	Egypt	Prospective (*n* = 82)	IFN-α	SLAM, SLEDAI	High IFN is correlated with increased disease activity, refractory to therapy, and lupus nephritis
Fu 2008 (Fu et al., 2008)	China	Cross-sectional (*n* = 67)	IFN signature	SLEDAI, SLICC, lupus nephritis	Increased gene signature is associated with worse SLEDAI, SLICC, and prevalence of lupus nephritis
Guthridge 2020 (Guthridge et al., 2020)	United States	Cross-sectional (*n* = 198)	IFN Signature	SLEDAI, lupus nephritis	Increased IFN signature is associated with increased SLEDAI and renal activity
Han 2020 (Han et al., 2020)	United States	Cross-sectional (*n* = 141)	IFN signature	Leukopenia	Increased interferon signature is associated with leukopenia
Iwamoto 2022 (Iwamoto et al., 2022)	United States	Cross-sectional (*n* = 221)	IFN signature	Lupus Nephritis	Type I IFN is a strong predictor of class III/IV lupus nephritis
Jakiela 2018 (Jakiela et al., 2018)	Poland	Cross-sectional (*n* = 35)	Urinary IFN signature	Lupus Nephritis	Patients with active lupus nephritis had increased urinary cytokines
Landolt-Marticorena 2009 (Landolt-Marticorena et al., 2009)	Canada	Prospective (3 months to 1 year) (*n* = 94)	IFN signature	SLEDAI	Baseline increased interferon signature is associated with increased disease activity—however, changes in interferon signature are not associated with clinical outcomes
Mai 2021 (Mai et al., 2021)	Canada	Prospective (*n* = 137)	IFN Signature	SLEDAI, SLE Flare Index	High baseline interferon index is associated with more severe disease activity and flares
Merrill 2017 (Merrill et al., 2017)	United States	Prospective (6 months) (*n* = 41)	IFN Signature	SLEDAI, BILAG	Patients with high IFN signature had improvement in IFN after treatment with HCQ. Unclear if this clinically correlated
Munroe 2017 (Munroe et al., 2017)	United States	Prospective (3 months) (*n* = 31)	IFN signature	Disease flare	Increased baseline IFN signature is associated with an increased risk of disease flare
Northcott 2022 (Northcott et al., 2022)	United States	Prospective (*n* = 205)	IFN signature	SLEDAI	Baseline high IFN status is associate with more severe disease but does not change over time
Petri 2019 (Petri et al., 2019)	United States	Prospective (2 years) (*n* = 243)	IFN signature	SLEDAI, serologic markers	Increased baseline IFN signatures were associated with increased skin and arthritis—however, changes in IFN signatures were not associated with changes in disease activities
Rose 2013 (Rose et al., 2013)	Germany	Prospective (6 months) (*n* = 79)	IFN Signature	SLEDIA, BILAG	Increased levels of IFN- α are associated with increased disease activity
Schneider 2015 (Schneider et al., 2015)	Brazil	Cross-sectional (*n* = 172)	IFN signature	SLEDAI	Increased baseline IFN is associated with increased SLEDAI.
Wilkinson 2020 (Wilkinson et al., 2020)	International Multicenter	Post hoc meta-analysis from Phase 3 Belimumab RCTs (*n* = 554)	IFN-1 signature	SRI-4 response rate	Patients with increased baseline IFN signatures had improves response rates compared to their counterparts
Willis 2012 (Willis et al., 2012)	United States	Prospective (1 year) (*n* = 35)	IFN signature	SLAM-R	Patients with increased IFN signatures had increased disease activity. Patients with improved disease activity after hydroxychloroquine therapy had decreased IFN-a

Abbreviations: SLE, systemic lupus erythematosus; IFN, interferon; SLAM, systemic lupus activity measure; SLEDAI, SLE disease activity index; SLICC, SLE international collaborating clinics damage index; BILAG, british isles lupus assessment group; SLE response index (SRI-4) response rate.

There has also been interest in determining if IFN levels may help predict the response to treatments in SLE patients. Adel et al reported that serum levels of IFN-α were associated with the presence of lupus nephritis and a poor response to immunosuppressive treatments (Adel and Sadeq, 2020). However, a meta-analysis of randomized controlled phase 3 trials of belimumab in the treatment of SLE, including 554 SLE patients found that patients with increased baseline levels of IFN were more likely to respond to therapy with belimumab than those with lower baseline IFN levels (Wilkinson et al., 2020). In a pooled post-hoc analysis of both the TULIP-1 and TULIP-2 randomized controlled trials testing the clinical effectiveness of the anti-IFNAR monoclonal antibody anifrolumab (discussed below), SLE patients with higher baseline interferon levels had better response rates to anifrolumab treatment (Vital et al., 2022). This was also noted in the phase 2 trials with anifrolumab (Furie et al., 2017). However, this data should be interpreted with caution as it remains unclear whether the improved responses to therapy in these trials are because patients at baseline had an increased disease activity, and therefore more likely to improve. Furthermore, the difference in the ability of type I IFN activity to predict treatment responses might also depend on the mechanism of action of the intervention. Therefore additional prospective studies are needed to better understand the predictive capacity of baseline type I IFN activity with treatment responses.

The increased expression of type I IFNs also associated with specific disease manifestations including lung disease, lupus nephritis, skin disease, arthritis, and leukopenia (Al-Mutairi et al., 2007; Fu et al., 2008; Petri et al., 2019; Adel and Sadeq, 2020; Han et al., 2020). A recent study found the presence of class III or IV lupus nephritis was increased in patients with higher expression of the type I IFN, assessed using an IFN bioassay (OR 5.4, *p* < .01) (Iwamoto et al., 2022). Furthermore, the IFN signature was more predictive for lupus nephritis than low complement C3 levels and anti-double stranded DNA antibodies. Increased urinary excretion of type IFNs has also been associated with lupus nephritis (Jakiela et al., 2018). However, these sample sizes have been small and have not been adequately controlled to draw further recommendations for clinical practice. Finally, cerebrospinal levels from patients with neuropsychiatric lupus had elevated levels of IFN-α compared to patients with other neurological diseases such as multiple sclerosis (Santer et al., 2009).

Together these studies highlight that type I lFN are associated with active lupus manifestations and suggest a potential use of the IFN signature to stratify patients into those with higher and lower risk of developing future severe disease activity and manifestations, and possible predict treatment responses. However more studies are needed to validate these data and to determine how precisely this may be utilized by practitioners.

### Trending type I IFN levels with SLE disease activity

The use of the IFN levels or gene expression has also generated interest if levels can be followed over time and used as a biomarker of disease activity. For example, two studies compared the levels of IFN in SLE patients with a high baseline level that initiated treatment with hydroxychloroquine (Willis et al., 2012; Merrill et al., 2017). After initiating treatment, patients had a significant decrease in IFN levels, and improvement in disease activity. Interestingly, this was not seen with other immunosuppressants such as azathioprine. However, a treatment effect on type I IFN levels was not observed in a larger study (Petri et al., 2019). In this cohort, while patients with a high baseline IFN level had increased disease activity, the type I interferon scores were stable over a 2 year period including, after treatment there. Therefore, there was no correlation between IFN levels and improvement in disease activity (Petri et al., 2019). Finally, other studies have confirmed that IFN signature is stable across time and does not correlate with changes in disease activity, except for high doses of glucocorticoids (Northcott et al., 2022). It is not currently known why interferon expression is stable and does not correlate with SLE activity in patients who have an inherently high type I IFN expression. It has been suggested that genetic factors may drive the increase in type I interferons as opposed to disease activity, but additional research is needed better understand these questions. Together these studies suggest that while baseline type I IFN activity might predict future disease activity, their role in tracking disease activity and response to treatment is likely limited.

Finally, our understanding of the relationship of long-term outcomes in SLE patients and type 1 IFN gene signatures remains poor. Fortunately, there is an ongoing 3 year observational study following patients with low and high IFN gene signatures with plans to enroll 900 patients. The estimated study completion is 2022 and will hopefully provide substantial additional insight in the application of type I IFN activity as a SLE biomarker (Hammond et al., 2020).

## Therapeutic targeting of type I interferons in SLE

### Anti-IFN therapy and SLE

The critical role of type1 IFNs in SLE immunopathogenesis makes it an intriguing therapeutic target in SLE. Multiple biologics have been developed targeting the type1 IFN pathway as well as a unique anti-IFNα vaccine strategy. These studies have produced conflicting results, perhaps due to the large number of type1 IFN with potentially redundant functions. These studies are summarized in Table 2.

**TABLE 2:** Randomized Controlled Clinical Trials of anti-interferon therapy.

Study	Trial phase	Number of participants	Clinical efficacy (primary endpoints)	Serious adverse events
Anifrolumab				
Furie 2017 (Furie et al., 2017)	2	305	Patients with anifrolumab had improved SRI and BICLA scores *versus* placebo	Anifrolumab patients had more infections with Herpes zoster
Tanaka 2020 (Tanaka et al., 2020)	2	20	This study was not powered for clinical efficacy	Adverse events were similar between anifrolumab and placebo
Jayne 2022 (Jayne et al., 2022)	2	147	Similar clinical outcomes for lupus nephritis between anifrolumab and placebo	Increased Herpes Zoster in anifrolumab *versus* placebo
Furie 2019 (Fure et al., 2019)	3	457	There was no difference in SRI-4 response rates between Placebo and Anifrolumab. However, patients had improved CLASI scores and BICLA responses	There were similar serious adverse events between anifrolumab and placebo
Morand 2020 (Morand et al., 2020)	3	362	Anifrolumab had improved BICLA and SRI scores than placebo	Anifrolumab patients had increased infections with Herpes zoster
Sifalimumab				
Merrill 2011 (Merrill et al., 2011)	1	33	Less disease flares as measured by SLEDAI	Similar between placebo and Sifalimumab
Petri 2012 (Petri et al., 2013)	1	161	No differences in clinical outcomes between placebo and Sifalimumab	Similar between placebo and Sifalimumab
Khamashta 2016 (Khamashta et al., 2016)	2	431	Patients with Sifalimumab had improved SRI scores than placebo	There was increased Herpes zoster infections with Sifalimumab *versus* placebo
Takeuchi 2020 (Takeuchi et al., 2020)	2	30	Not powered for clinical efficacy	There was no placebo group, but Sifalimumab was well tolerated
Rontalizumab				
McBride 2012 (McBride et al., 2012)	1	60	Not powered to detect clinical efficacy between both groups	Similar adverse events between placebo and rontalizumab
Kalunian 2016 (Kalunian et al., 2016)	2	238	No difference between placebo and rontalizumab	Similar adverse events between placebo and rontalizumab
IFN-α Kinoid				
Houssiau 2019 (Houssiau et al., 2020)	2	185	No differences noted between placebo or IFN-k in the primary endpoints	Similar adverse events between IFN-k and placebo

Abbreviations: SRI-4, SLE response index-4; BICLA, BILAG-based composite lupus assessment; CLASI, cutaneous lupus erythematosus disease area and severity index; SLEDAI, SLE disease activity index.

### Sifalimumab

Sifalimumab is a monoclonal antibody that directly targets multiple subtypes of IFN-α. This drug was studied in phase 1 and 2 trials (Merrill et al., 2011; Petri et al., 2013; Khamashta et al., 2016; Takeuchi et al., 2020). There has been one published successful phase 2 trial for the use of sifalimumab in patients with SLE (111). This trial enrolled 431 patients with SLE and randomized these patients into varying doses of sifalimumab (200 mg, 600 mg, or 1200 mg) and placebo. The primary endpoint for this study was SLE Responder Index 4 (SRI-4) response rate and found that patients on sifalimumab had improved disease activities (59.8% 1200 mg, 56.5% 600 mg, 58.3% 200 mg, 45.4% placebo). Despite the promise this drug showed in phase 2 trials (Khamashta et al., 2016), its development was discontinued in favor of anifrolumab, which showed greater efficacy in pre-phase 3 trial data as discussed below.

### Rontalizumab

Rontalizumab is a monoclonal antibody that directly binds to multiple subtypes of IFN-α. Trials for this agent also did not show clinical efficacy including a phase 2 trial with 238 patients that were randomized to either 750 mg or 300 mg of rontalizumab *versus* placebo (Kalunian et al., 2016). This study showed that there were no differences in responses in the primary outcomes (SRI-4 or BILAG-based Composite Lupus Assessment (BICLA) responses). Due to this negative study, drug development was discontinued.

### Interferon- α kinoid

IFN-α kinoid (IFN-k) is an interesting immunotherapeutic vaccine agent in which patients are injected with IFN-k and subsequently develop neutralizing anti-IFN antibodies. There has been one phase IIb trial conducted for IFN-k (Houssiau et al., 2020). This study enrolled 185 patients who were randomized into either IFN-k *versus* placebo. This study did not meet the primary endpoint of BICLA response rate but did show improvements in SRI-4 response rates and attainment of lupus low disease activity states. There have been no phase 3 trials registered for this therapy.

### Anifrolumab

Anifrolumab is a monoclonal antibody that binds to IFNAR, therefore blocking the activity of all type I IFN. Anifrolumab is the only anti-IFN therapy to undergo Phase 3 randomized controlled trials (RCTs). The RCTs for Anifrolumab are summarized in Table 2. There have been three large double-blinded RCTs published for anifrolumab–the MUSE, TULIP-1, and TULIP-2 trials (Furie et al., 2017; Fure et al., 2019; Morand et al., 2020).

The MUSE was a multicenter phase 2b trial that randomized patients into varying doses of anifrolumab (300 mg or 1000 mg) and placebo. A total of 307 patients were enrolled. The primary outcome for this trial was the SRI-4 at 6 months with sustained reduction in corticosteroids. This study met its primary end point of improved SRI-4 response rates (36% anifrolumab 300 mg, 28% anifrolumab 1000 mg, 13% placebo). Furthermore, this trial demonstrated that SLE patients with a baseline high IFN signature had the largest treatment response. There were also improvements in BICLA responses, tender joints, and Cutaneous Lupus Erythematosus Disease Area and Severity Index (CLASI) scores.

The TULIP-1 was a phase 3 double-blinded RCT that randomized patients to anifrolumab (300 mg or 150 mg) or placebo. A total of 457 patients were enrolled in this trial. The primary endpoint was SRI-4 responses. This trial failed to meet its primary outcome at 1 year (36% response in anifrolumab *versus* 40% response in placebo, 95% CI -14.2 to 5.8). However, secondary outcomes such as BICLA response, CLASI scores, joint tenderness, and corticosteroid reduction were observed with anifrolumab. The authors of this study hypothesized that the BICLA response rate but not the SRI-4 met the primary outcomes because the BICLA response rate can capture partial improvements in disease activity. Furthermore, they attributed the failure of reaching the primary outcome due to strict outcomes, such as considering the use of non-steroids anti-inflammatory drugs (NSAIDs) as a treatment failure for SLE.

The TULIP-2 trial was a phase 3 double-blinded RCT that randomized patients to anifrolumab 300 mg and placebo. There were 365 patients enrolled. Due to the failure of the TULIP-1 not meeting its primary endpoint of SRI-4 response, the endpoint of TULIP-2 was modified to the BICLA response to capture partial improvements. This study showed an increased BICLA response for the anifrolumab *versus* placebo groups (48% *versus* 32%, *p* < .05). Furthermore, the anifrolumab patients had improvements in the SRI-4, CLASI scores, and corticosteroid reduction. Interestingly, there were no differences noted in swollen and tender joints.

These studies overall indicate that anifrolumab is an agent that has efficacy for cutaneous and musculoskeletal activity for patients with SLE. Furthermore, it has been shown in these studies to be a useful agent to specifically help patients titrate down glucocorticoids. Accordingly, anifrolumab was granted FDA approval for the treatment of SLE on 30 July 2021. However, to date, the use of anifrolumab has not been adequately studied in severe organ manifestations such as lupus nephritis but these studies are currently being performed. There has been a recently published phase 2 trial for the use of anifrolumab in patients with lupus nephritis (Jayne et al., 2022). Unfortunately, this study did not meet its primary endpoint. However, it was hypothesized that patients with lupus nephritis have increased excretion of anifrolumab related to their proteinuria and therefore at “baseline dosing” may have inadequate serum levels to achieve a therapeutic effect. In the patient population that was treated with higher doses of anifrolumab, there were improvement in surrogate markers suggesting a potential therapeutic role for high doses of anifrolumab. Currently anifrolumab is being tested in an ongoing phase 3 clinical trial in lupus nephritis (AstraZeneca, 2022).

Adverse reactions from anifrolumab in phase 3 trials were primarily from herpes zoster infections and upper respiratory tract infections (including bronchitis and pneumonias). These are hypothesized to be due to decreased antiviral responses that require interferon to be activated. There were also severe hypersensitivity reactions noted. Currently, there is insufficient data to determine if anti-IFN therapies have an increased risk of malignancy.

## Alternative therapeutic strategies targeting type I IFN in SLE

As noted above, there are multiple steps in the type I IFN pathway that are activated and play a role in SLE. Accordingly, the type I IFN pathway may be therapeutically targeted in multiple ways, aside from directly targeting IFN-α or IFNAR. For example, as noted in Figure 1, type I IFN signal through JAK proteins. Targeting JAK proteins has been successful in the treatment of rheumatoid arthritis (Burmester et al., 2013; Genovese et al., 2016; Genovese et al., 2018). Baracitinib, a JAK1 and JAK2 inhibitor, has been tested in a phase 3 trial, demonstrating improvements of SRI-4 (BRAVE 1) but a separate phase 3 trial did not show similar improvements (BRAVE II) (Updates, 2022). As such, this drug’s investigation in SLE has been suspended. Upadacitinib, a selective JAK1 inhibitor, is currently being investigated in a phase 2 clinical trial in combination with Elsubrutinib–a Bruton tyrosine kinase inhibitor. The phase 2 trial has been completed and results are currently pending.

Finally, as noted above, pDCs are critical producers of type I IFN. As such, directing therapies against pDCs is a potential strategy to target type I IFN in SLE. Litifilimab is a humanized IgG1 targeting BDCA2 receptor on pDCs, which subsequently decreases type I IFN levels. A recent phase 2 clinical trial with 334 patients showed improvements in tender and swollen joints *versus* placebo (Furie et al., 2022). However, secondary endpoints including disease activity markers and skin disease showed no differences (Furie et al., 2022). Interestingly, a separate phase 2 trial investigating litifilimab for cutaneous SLE found improvements in CLASI scores *versus* placebo (Werth et al., 2022). Both these trials showed an increase in herpes zoster infections. Additional studies are needed to determine the efficacy of litifilimab in SLE.

### Future directions

Although anti-interferon therapies, especially anifrolumab, have shown promise in SLE, there are still many unanswered questions. More studies are needed to determine if anifrolumab will be efficacious in more severe manifestations of SLE such as nephritis and there are on going trials testing anifrolumab in the treatment of lupus nephritis. More evidence is also needed to determine the role of anifrolumab in subsets of patients (ex: males *versus* females, younger *versus* older, different racial groups). Current evidence shows that type 1 interferons may be expressed higher in females *versus* males (Ziegler et al., 2017; Webb et al., 2018). This suggests that anti-interferon therapy may also have a stronger effect in males *versus* females, though this is not currently known. Another important consideration is understanding when anifrolumab should be used in SLE patients, as opposed to other therapeutics, especially biologics. There is no evidence as to how anifrolumab may directly compare to belimumab (another FDA approved biologic for the treatment of SLE) as there have been to head-to-head trials. However, anifrolumab showed marked improvement in cutaneous manifestation of SLE in the TULIP-1 and TULIP-2 trials (Fure et al., 2019; Morand et al., 2020). However, the role of belimumab in cutaneous SLE has not been as extensively tested in trials. In the future, it may be that the decision of anifrolumab *versus* belimumab may be decided through disease manifestations (i.e., anifrolumab for cutaneous SLE and belimumab with mycophenolate for lupus nephritis), but these treatment decisions will require future studies to better inform the clinicians and patients.

Lastly, it is also of interest to see if type I interferon therapeutics will be beneficial in the treatment of other autoimmune diseases with activation of the type I interferon pathways. Type I interferons have been shown to be elevated in patients with Sjögren’s syndrome (Wildenberg et al., 2008; Vakaloglou and Mavragani, 2011; Bodewes and Versnel, 2018), systemic sclerosis (Assassi et al., 2010), mixed connective tissue disease (Paradowska-Gorycka et al., 2021) and dermatomyositis (Baechler et al., 2007; Pinal-Fernandez et al., 2019). A phase 1 b study in dermatomyositis patients was conducted with sifalimumab, which suggested a small benefit (Higgs et al., 2014). Non-controlled, pilot clinical studies a have suggested that JAK inhibitors, which can block type I interferon signaling, might be beneficial in adult and pediatric cases of dermatomyositis (Ladislau et al., 2018; Le Voyer et al., 2021; Paik et al., 2021). Finally, anifrolumab is also currently being investigated in a phase II trial for the treatment of Sjögren’s Syndrome (NCT05383677, ANISE-II). While these studies are of interest, larger, randomized controlled clinical trials are needed to determine if targeting type I interferons is a viable strategy in these autoimmune diseases as it has been shown to be in SLE.

## Conclusion

The IFN pathways are highly involved in the pathogenesis in SLE and have provided excitement in the diagnosis, prognosis, and treatment of patients with SLE. However, more research is currently needed to understand the extent to which IFN gene signatures can be used in clinical practice. The emergence of anifrolumab highlights the potential for targeting type I IFN in the treatment of SLE, but continued studies of anifrolumab and other therapeutics targeting type I IFN are important to determine the extent to which targeting type I IFN will be used in the treatment of SLE.
